# Observations on the Use of Antigonococcic Serum with Report of Cases

**Published:** 1909-08

**Authors:** W. C. Danforth

**Affiliations:** Evanston, Ill.


### CLINICAL REPORT

### Observations on the Use of Antigonococcic Serum with Report of Cases

By W. C. DANFORTH, M. D., Evanston, Ill.

CASE I.—R. F. A., age 38 years; male; married; clerk. Had acute gonorrheal urethritis six weeks ago; was treated by home physician; discharge stopped. About two weeks later he began to have rheumatic pains in the left knee, also in the shoulder, and soon after in the right knee; pain in the knees accompanied by swelling; was unable to use legs.

Status presens: pain in both knees, especially the left one, and the patient cannot use his lower extremities except to walk very slowly with assistance; some pain in the right lumbar region and tenderness on pressure; left knee greatly swollen; signs of fluid plainly elicited; tenderness on pressure; some spontaneous pain; same signs in less degree on the right side; much pus in the urine; temperature, 100° to 101.8°. Diagnosis, gonorrheal arthritis.

*Treatment.*—Knees aspirated twice. Oleate of mercury in unctions; pressure bandages. Only slight improvement from this procedure. Injections of antigonococcic serum (P., D. & Co). were made as follows: December 26, 1907, 2 cc.; December 28, 2 cc.; December 30, 2 cc.; January 1, 1908, 2 cc.; January

3, 2 cc.; January 5, 2 cc.; January 7, 2 cc.; January 9, 2 cc.; January 11, 2 cc.; January 13, 2 cc.; January 15, 2 cc.

After the second injection the patient stated that he felt distinctly more comfortable; the pain in the knees was much easier. After the fourth injection the effusion seemed to have ceased to accumulate and the temperature had declined. After the sixth injection the patient was able to walk with comparative comfort. Examination after five months showed complete recovery.

CASE II.—D. H.; age, 30 years. Male; single; salesman. Had had several attacks of gonorrheal urethritis during the past ten years. For three or four years there had existed a posterior urethritis—almost always with some slight discharge, the urine contained shreds and gonococci microscopically demonstrable; three months ago another acute attack supervened. The patient complained of pain at the base of the bladder with frequent urination; the general health was much below par; he also complained of weakness.

On examination I found a moderate amount of discharge; gonococci present; prostate slightly large and distinctly tender; the patient was anemic and had been confined to bed for three days.

The treatment embraced the usual routine measures, local and internal. Antigonococcic serum, P., D. & Co., was administered as follows: January 23, 1908, 2 cc.; January 25, 2 cc.; January 29, 2 cc.; January 31, 2 cc.; February 2, 1908, 2 cc.; February 6, 2 cc.; February 10, 2 cc.

After three injections the patient stated that he felt distinctly better and he went back to work one week after the commencement of treatment. The amount of discharge diminished and the prostatic symptoms disappeared.

February 6, 1908, the patient reported that he was much pleased with his changed physical condition; he stated that he felt distinctly stronger and better. The discharge was very slight. Gonococci microscopically demonstrable in small numbers. February 10, 1908, very few gonococci found. The patient feels very well and is actively engaged in business.

CASE III.—Mr. W.; age 21 years. Single; clerk. Had gonorrhea one month before calling on me. For the past two weeks there had existed a swelling with pain in the left knee, wrist and little finger; he could use the wrist and hand but slightly and walked with great discomfort.

At the time of his visit great pain was evident in wrist and finger with tenderness on pressure and immobility of affected joints. The knee also was tender and motion limited; he walks with great discomfort. The knee was much distended with fluid. Diagnosis, gonorrheal arthritis.

*Treatment.*—Antigonococcic serum, 2cc. This dose was repeated every second day until six injections had been made; then there was an intermission of one week, followed by three more injections of the same dose at similar intervals. September 10, by aspiration of the left knee, three ounces of turbid sero-pus was removed. September 23 aspiration of the left knee removed two ounces of fluid that was considerably clearer than the former specimen.

After the first three injections of antigonococcic serum the discomfort was markedly decreased. After six injections the patient was entirely comfortable; the swelling of the wrist was much reduced; after a week's intermission there were some signs of recurrence in the knee. After nine injections the patient admits that he is practically cured.

*Present condition.*—Wrist freely movable; skin no longer tense and drawn; the patient can perform any movement almost to the normal extent. He is now at work and walks without limp and with no discomfort. There is no doubt that these cases, particularly those with arthritic symptoms, were greatly benefited by the use of antigonococcic serum, the improvement being noted within a short time after commencing its use. The first case noted was more than usually severe and had shown no signs of improvement up to the time the use of antigonococcic serum was instituted.

University Building.

